# Clinical and Histological Findings of Male Uterus (*Uterus Masculinus*) in Three Dogs (2014–2018)

**DOI:** 10.3390/ani13040710

**Published:** 2023-02-17

**Authors:** Giorgia Tura, Giulia Ballotta, Marco Cunto, Massimo Orioles, Giuseppe Sarli, Daniele Zambelli

**Affiliations:** 1 Department of Veterinary Medical Sciences, University of Bologna, Via Tolara di Sopra 50, Ozzano dell’Emilia, 40064 Bologna, Italy; 2 Veterinary Pathology Unit, Department of Agricultural, Food, Environmental and Animal Sciences, Via Sondrio 2, 33100 Udine, Italy

**Keywords:** *uterus masculinus*, imaging ultrasound, histology, dog

## Abstract

**Simple Summary:**

In this study, we report the clinical and histopathologic diagnostic workup of three cases of *uterus masculinus,* the Müllerian duct remnant, in dogs. In the literature, single case reports describe this pathology in the canine species and, among other domestic mammals, in the horse. Its definitive diagnosis requires a specialized approach by integrating diagnostic imaging, surgery and histopathology analysis; furthermore, inflammation of *uterus masculinus* in the dog should be listed as a differential cause of abdominal pain.

**Abstract:**

Data from three cases of *uterus masculinus* were retrieved from 2014 to 2018. Two out of three cases presented clinical signs compatible with systemic infection, as observed in bitches with pyometra. Ultrasound examination revealed a tubular fluid-filled structure with a thin irregular wall located cranially to the prostate and in continuity with the cranial part of the gland. In two cases, two other tubular fluid-filled structures were visualized in the caudal part of the abdominal cavity, ventrally to the prostate gland and urinary bladder. After surgical removal of these, histological examination revealed the presence of a uterine structure morphologically similar to the female counterpart. Various types of epithelial cell lining were found, including simple columnar, simple stratified and squamous epithelium associated with glands in the underlying stroma. Immunohistochemistry to anti-Müllerian hormone (AMH) produced a positive result on glands, while multifocal expression was found in the lining epithelium. AMH seems involved in the pathogenesis of *uterus masculinus*, but its role is not fully understood. Thorough clinical and ultrasonographical examinations, followed by a histological confirmation, are necessary to properly diagnose *uterus masculinus* in dogs.

## 1. Introduction

*Uterus masculinus*, also called prostatic utricle, is thought to be the remnant of the Müllerian duct, which is present in both sexes during the embryonic period. During embryogenesis, it differentiates into tubular genitalia in females, while in males, it regresses after testicular development [1]. Between the 36th–46th day of canine gestation, functional Sertoli cells of the fetal dog testis produce anti-Müllerian hormone (AMH), a glycoprotein belonging to the TGF-β superfamily secreted by Sertoli cells in fetal testes [2], but in male dogs also by a Sertoli cell tumor [3], that inhibits the development of the Müllerian duct. At the same time, Leydig cells produce testosterone, which induces the differentiation of Wolffian ducts and the development of epididymides, vasa deferentia and seminal vesicles [1,4].

The pathogenesis of *uterus masculinus* is not fully understood, but absent or non-functional anti-Müllerian hormone (AMH) receptors seem to be involved [5].

Among congenital anomalies of the reproductive tract, persistent Müllerian duct syndrome (PMDS) is the less-extreme and best-recognized form of disorder of sexual development in dogs [1]. This syndrome is characterized by Müllerian derivatives in the normally masculinized XY genotype. Canine PMDS and the associated clinical signs have been largely reported in miniature schnauzer [6,7]. A single homozygous cytosine substitution to thymidine in exon 3 at nucleotides 241 of the MISRII gene was identified as the causative genetic defect in this breed [8]. Affected dogs can present with an oviduct, uterus, cervix and cranial vagina. Bilateral or unilateral cryptorchidism may arise as clinical sequelae of the disease, sometimes associated with infertility and increased risk of testicular neoplasms [9,10].

In human medicine, the presence of *uterus masculinus* is well described and associated with variable clinical presentation ranging from asymptomatic to recurrent urinary tract infection, epididymitis, hematuria, pyuria, urinary incontinence, oligospermia, retention or constipation [11,12]. In veterinary medicine, few cases have been reported in small and large animals. In large animals, it is often described as an occasional finding, especially in horses, displayed as a cystic cul-de-sac structure within the colliculus seminalis. Its presence is asymptomatic and only detected if the cysts enlarge and compress ejaculatory ducts, leading to ejaculatory dysfunction, poor quality of semen and discomfort [13,14]. In dogs, *uterus masculinus* has been observed to be associated with dysuria, hematuria, purulent preputial discharge, urinary tract infection, prostatitis, cryptorchidism and testicular neoplasia (especially Sertolioma) [9,15,16,17]. A case of *uterus masculinus* neoplasia has been reported recently in a dog [3]. *Uterus masculinus* has been described as a single pouch or as a bi-horned structure resembling a true female uterus [18,19,20]. In most cases, it is asymptomatic and can be occasionally detected as an incidental finding during routine ultrasonography or surgery [19]. Commonly, it can be misdiagnosed as paraprostatic cysts because of its anatomical position and its cystic appearance [18].

This retrospective study aimed to describe the histological and clinical features of three cases of *uterus masculinus*.

## 2. Materials and Methods

The medical records of the Department of Veterinary Medical Science were searched for cases with a diagnosis of *uterus masculinus* from 2014 to 2018. Clinical findings and signalment data were retrieved. Histological samples were available as formalin-fixed, paraffin-embedded sections stained with hematoxylin and eosin.

History

A 6-year-old unilateral cryptorchid male miniature schnauzer (case 1) was referred for the presence of a tubular fluid-filled structure in the scrotum. The dog was orchiectomized for unilateral cryptorchidism associated with testicular neoplasm (of unknown histotype). Exploratory laparotomy revealed a structure similar to a fluid-filled uterus and a normal prostate.

An 8-year-old neutered male mongrel dog (case 2) was referred for abdominal swelling. The dog was neutered one month before for testicular neoplasm (even though the histotype of the tumor was unknown). At the ultrasound examination, a suspicion of *uterus masculinus* or paraprostatic cyst arose.

A 6-year-old basset hound (case 3) was presented for a second opinion considering that the dog was an important sire, and orchiectomy was proposed as a therapy for orchitis. The andrological examination highlighted a bilateral testicular neoplasm associated with prostatic squamous metaplasia and an infected paraprostatic cyst. Fine-needle aspiration of the prostatic gland and cytology of the preputial mucosa were additionally performed.

All ultrasound examinations were performed with Esaote MyLab Vet5 using a 6.5–15 MHz micro convex probe.

In all dogs, biological samples resected during the surgical procedure, including testes for cases 1 and 3, were submitted for histological study. Briefly, samples were fixed in formalin and embedded in paraffin. Paraffin-embedded tissues were cut into consecutive three-micron-thick sections and stained either with hematoxylin–eosin (H&E; hematoxylin cat no. 01HEMH2500; eosin cat no. 01EOY101000; Histo-Line Laboratories, Antigliate, MI, Italy) and immunohistochemistry to AMH. Three-micron-thick sections of each sample were dewaxed and rehydrated for this latter stain. Endogenous peroxidase was blocked by immersion in 3% H_2_O_2_ in methanol for 30 min at room temperature (RT). Antigen retrieval (three cycles of incubation in a pH 8.0 EDTA buffer heated for 20 min in a microwave oven at 750 W) was followed by cooling at RT for 20 min. Blocking of non-specific antigenic sites was achieved by incubating the slides in a solution of 10% normal goat serum in PBS (blocking solution) for 30 min at RT and afterward incubating them overnight in a humid chamber at 4 °C with the primary antibody (MIS clone B-11 Santa Cruz Biotechnology, Santa Cruz, CA, USA) diluted 1:30 in blocking solution.

The slides were rinsed in TRIS buffer and then incubated for 30 min RT with a secondary anti-mouse antibody diluted 1 in 200 in blocking solution. After two washes in TRIS buffer, immunoreactions were detected with avidin–biotin immunoperoxidase (Vectastain Elite ABC Kit, Vector Laboratories, Burlingame, CA, USA) and visualized with the chromogen 3,3′-diaminobenzidine (0.05% *w*/*v*, cat# ACB999, Histo-Line Laboratories). Slides were counterstained with Harris hematoxylin (Histo-Line Laboratories) and permanently mounted with DPX medium (Fluka, Riedel-de Haen, Germany).

Slides were observed with a Nikon Eclipse E600 microscope (Nikon Instruments Europe BV, Amsterdam, The Netherlands) equipped with the Imaging Source “33” Series USB 3.0 Camera (DFK 33UX264; Bremen, Germany).

## 3. Results

### 3.1. Clinical Data

**Figure 1:**
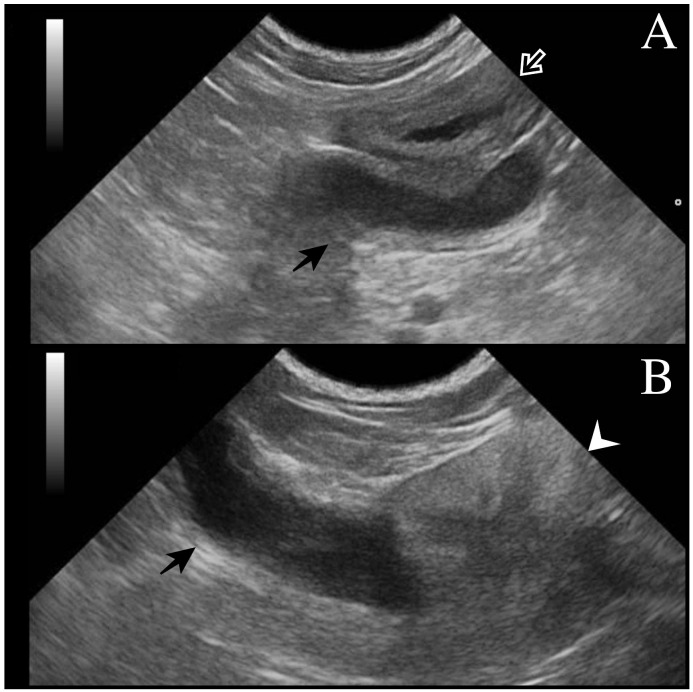
Transverse ultrasonographic image of *uterus masculinus*: (**A**) Black arrow points to the *uterus masculinus* filled with hypoechoic fluid and localized near the urinary bladder (white arrow). The wall of the *uterus masculinus* is isoechoic to the urinary bladder wall. (**B**) *Uterus masculinus* with a fluid-filled lumen (black arrow) originating from the cranial part of the prostate gland (white arrowhead) and extending cranially.

**Figure 2:**
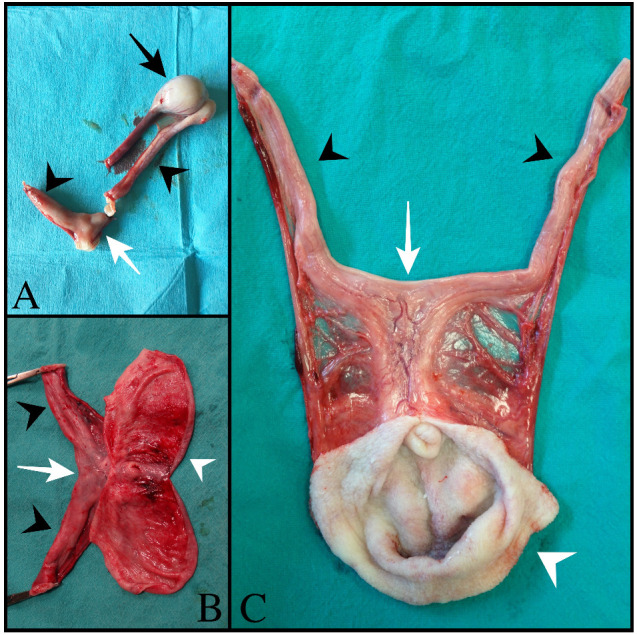
Gross anatomy of *uterus masculinus*: (**A**) Case number 1: The black arrow points to the normal testis. (**B**) Case number 2: White arrowhead points to the tubular fluid-filled structure originating from the dorso-cranial portion of the prostate gland. (**C**) Case number 3: White arrowhead points to the tubular fluid-filled structure originating from the dorso-cranial portion of the prostate gland. In all images, the black arrowhead points to the uterine horns, which arise from the body of the *uterus masculinus* (white arrow).

All dogs underwent a complete assessment supplemented with laboratory and ultrasonographic abdominal examination. At presentation, case 1 was asymptomatic, and the tubular fluid-filled structure in the scrotum was detected occasionally by the referring veterinarian during a routine consultation. Cases 2 and 3 presented clinical signs compatible with systemic infection (fever, depression, disorexia/anorexia, vomiting) as described in bitches during pyometra. Except for case 1 (no alterations detected), neutrophilic leukocytosis was observed from the hematological and serum chemistry profiles. In all cases, ultrasound examination revealed a tubular fluid-filled structure with a thin irregular wall located cranially to the prostate and in continuity with the cranial part of the gland (Figure 1). In cases 1 and 2, two other tubular fluid-filled structures were visualized in the caudal part of the abdominal cavity, ventrally to the prostate gland and urinary bladder. In case 3, prostatic metaplasia and preputial keratinization were indicative of hyperestrogenism. Explorative laparotomy was carried out in all dogs, and a bi-horned structure with a stalk connected to the dorso-cranial portion of the prostate gland was recognized. The structure extended caudally in the abdominal cavity engaging in the deep inguinal ring on each side; complete resection was performed in all cases, and in cases 1 and 3, the surgery was completed with orchiectomy. Macroscopically, the bi-horned structure was similar to a fluid-filled female uterus (Figure 2). Table 1 illustrates a comparison of clinical data from the three cases, including anamnestic and clinical details.

### 3.2. Histology and Immunohistochemistry

**Figure 3:**
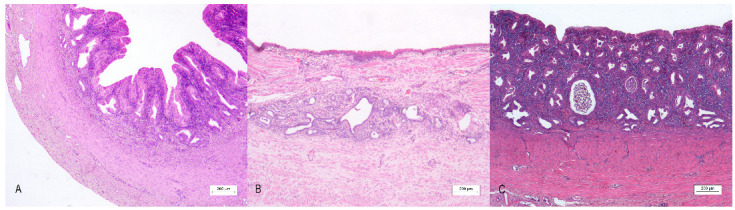
*Uterus masculinus*, histological findings: (**A**) Case number 3: The wall of the *uterus masculinus* with a three-layer appearance. Bar 200 µm. (**B**) Case number 2: Longitudinal section of the mucosal wall with squamous epithelium. Bar 200 µm. (**C**) Case number 1: Moderate lymphoplasmacytic and neutrophilic inflammation of the mucosal layer of the canine *uterus masculinus*. (**A**–**C**): Under 4× magnification, bar 200 µm, hematoxylin–eosin stain.

Histological features revealed the presence of a rudimental tubular structure with a three-layer appearance in all cases, resembling a uterine wall (Figure 3A). The innermost layer was composed of a simple columnar epithelium, with a multifocal microvacuolar cytoplasm and basal nucleus reminiscent of the progestinic change in the female uterus, supported by a moderate amount of fibrovascular stroma in cases 1 and 3. In case 3, luminal cells also exhibited an abrupt transition to a simple stratified epithelium, whereas in case 2, epithelial cells showed squamous metaplasia with multifocal keratinization (Figure 3C). Moderate lymphoplasmacytic and neutrophilic inflammation and numerous simple tubular glands filled with neutrophils and cellular debris were detected in all the cases (Figure 3B). The second layer consisted of densely packed smooth muscle cells resembling myometrium. An external thin layer of connective tissue represented the perimetrium. Histological evaluation from both testes of case 3 revealed the presence of an intratubular and invasive Sertoli cell tumor, while a testis of case 1 evidenced a normal testicular histology.

**Figure 4:**
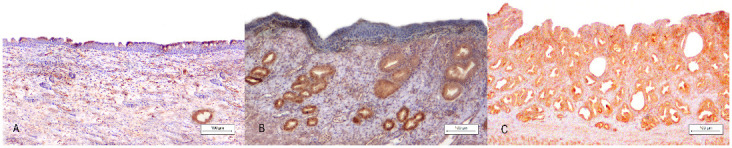
*Uterus masculinus*, immunohistochemical findings: (**A**) Case number 3: Multifocal-cytoplasmic AMH staining in the simple columnar epithelium of the uterine mucosa. Bar 100 µm. (**B**) Case number 2: Diffuse-negative immunolabelling of the uterine squamous epithelium and simultaneous strong glandular expression of AMH. Bar 100 µm. (**C**) Case number 1: Diffuse-strong glandular and epithelial AMH immunolabelling. (**A**–**C**): Under 10× magnification, bar 100 µm.

By immunohistochemistry, AMH was expressed in the cytoplasm of tubular glands in all cases. In contrast, luminal epithelial cells (Figure 4) showed diffuse-strong cytoplasmic positivity only in case 1, while the other two cases displayed multifocal AMH expression.

## 4. Discussion

Similar to previous reports, the *uterus masculinus* in this retrospective study involved older dogs. As described in the literature, all cases had a characteristic fluid-filled tubular structure cranially to the prostate [18,21].

Histological features of the three cases presented here were a wall formed by an epithelial lining and a muscular layer. The epithelial cell lining differed among the three cases, enhancing the variable appearance of this structure. In cases 1 and 3, a simple columnar epithelium with multifocal progestinic changes was present, supporting the evidence of a structure similar to the female uterus. Only case 2 exhibited squamous metaplasia of the lining epithelium. In the available literature, the histopathology of *uterus masculinus* shows various types of epithelial linings, including pseudostratified columnar, cuboidal and squamous epithelia [18,19,20,21]. The reason for this variability is not discussed in canine papers on the topic. Nevertheless, it can be argued that the uterus and the vagina develop from the caudal part of the Müllerian ducts, so both (respectively, simple columnar and squamous) linings can be present along their full length [22]. In addition, other variables that influence differentiation are estrogen and progesterone levels [21]. Although we do not have hormone levels for these three dogs, and we do not know the diagnoses of the testicular tumors in cases 1 and 2, squamous metaplasia induced by estrogen-producing Sertoli cell tumors may have been the reason for changes in the features of the epithelial lining.

In the cases described here, there was no clear distinction between the three muscle layers within the muscle wall, as seen in the female uterus. However, the data are not comparable with published papers as they are limited only to descriptions of the characteristics of the epithelial lining.

We further investigated the expression of AMH. In human medicine, AMH is commonly expressed in ovarian granulosa cells and endometrial cancer [6,7]. Immunolabelling of glandular and luminal epithelia is reported in a healthy human endometrium, with additional stromal positivity depending on the uterine functional phase [7,8]. The exact role of AMH in the human endometrium is not fully understood, but it seems to be involved in endometrial remodeling [8]. To date, no information is available on whether cells in the canine ovary or female reproductive tract express this protein. However, in all cases examined here, the AMH-positive stain, mainly in the endometrial glands of canine *uterus masculinus*, could be related to glandular remodeling.

In dogs, *uterus masculinus* is often reported in association with testicular tumors, especially estrogen-producing Sertolioma. High levels of circulating estrogen consistent with Sertoli cell tumors are thought to stimulate the development of the Müllerian duct system, leading to *uterus masculinus* [18,21]. In addition, in some cases, high progesterone levels were also detected, probably linked to the development of the glandular structure within the stroma [21]. Unfortunately, except for case 3, a complete endocrine evaluation was not possible as the other two dogs were previously orchiectomized. However, the simultaneous presence of progestinic changes in the columnar epithelium and glandular structures could have resulted from progesterone stimulation in cases 1 and 3. In the male organism, the synthesis of P4 is performed by the adrenal gland and the Leydig cells in the testicles [12]. In dogs, high levels of P4 are reported to be associated with a Sertoli cell tumor [23,24], and its production has been demonstrated in Leydig cells inside remnants of atrophic parenchyma [23].

Bilateral or unilateral cryptorchidism may arise as clinical sequelae of canine cases of persistent Müllerian duct syndrome (PMDS), with consequent infertility and an increased risk of testicular neoplasms [9,10]. Case 1, illustrated here, had unilateral cryptorchidism with a normal contralateral testis, according to previous studies’ findings [25]. Although genetic tests are currently available for PMDS, we did not perform any genetic analysis of the dog in case 1 due to the retrospective nature of the study.

## 5. Conclusions

This report suggests the importance of investigating the presence of *uterus masculinus* using ultrasonography and clinical examination in all dogs (symptomatic or asymptomatic) for which a specialist andrological examination is required or when an abdominal ultrasound is performed for any reason. Even if the diagnosis of asymptomatic *uterus masculinus* is uncommon, when available, preventive surgery should be considered in order to forestall inflammation of the structure.

The presence of variable histological features presented in these three cases should be considered and could be helpful for a proper diagnosis after surgery. Moreover, in human medicine, where *uterus masculinus* is well described in males, a few tumors, such as adenocarcinoma and uterine leiomyoma, have been observed to be related to this structure [26,27,28,29]. On the other hand, in veterinary medicine, only one case of adenocarcinoma of the *uterus masculinus* involving the prostate in a neutered Pomeranian dog has been described [3]. This finding emphasizes the importance of histological analysis to identify and characterize both the inflammatory state of the *uterus masculinus* and potential malignant changes of Müllerian remnants.

## Figures and Tables

**Table 1:** Anamnesis, symptoms at presentation and analogies/differences of ultrasonographic and laparotomic investigations in the three dogs with a final clinical diagnosis of *uterus masculinus*.

Case n.	Breed/Age	Anamnesis	Symptoms at Presentation	Ultrasonography		Explorative Laparatomy
1	Miniature Schnauzer 6-year-old	orchiectomy for unilateral cryptorchidism associated with ○ testicular neoplasm (unknown histotype) ○ tubular fluid-filled structure in the scrotum	asymptomatic	tubular fluid-filled structure located cranially to the prostate …	… and two other tubular fluid-filled structures, ventrally to the prostate gland and urinary bladder.	bi-horned structure with a stalk connected to the dorso-cranial portion of the prostate gland
2	Mongrel 8-year-old	neutered one month before for testicular neoplasm (histotype unknown)	abdominal swelling			
			fever, depression disorexia/anorexia vomiting neutrophilic leukocytosis			
3	Basset hound 6-year-old	orchitis			enlarged prostate with parenchyma heterogeneity	

## Data Availability

Not applicable.
